# Assessment of routine medical checkups for common noncommunicable diseases and associated factors among healthcare professionals in Addis Ababa, Ethiopia, in 2022 a cross-sectional study

**DOI:** 10.1097/MS9.0000000000000558

**Published:** 2023-04-01

**Authors:** Genanew Kassie Getahun, Meron Arega, Gebretsadik Keleb, Addis Shiferaw, Dawit Bezabih

**Affiliations:** a Kotebe Metropolitan University, Menelik II Medical and Health Science College; b Yanet health college; c Kea-med College, Addis Ababa; d School of Public Health, College of Medicine and Health Sciences, Jigjiga University, Jigjiga, Ethiopia

**Keywords:** Ethiopia, healthcare workers, noncommunicable diseases, routine medical checkup

## Abstract

Routine medical checkups are one strategy for detecting and treating noncommunicable diseases early. Despite the effort to prevent and control noncommunicable diseases in Ethiopia, the prevalence of the problem is significantly increasing. The aim of this study was to assess the uptake of routine medical checkups for common noncommunicable diseases and associated factors among healthcare professionals in Addis Ababa, Ethiopia, in 2022.

**Methods:**

A facility-based cross-sectional study was conducted, enroling 422 healthcare providers in Addis Ababa. A simple random sampling method was used to select study participants. Data entry was made using Epi-data and exported to STATA for further analysis. A binary logistic regression model was used to determine predictors of routine medical checkups. In the multivariable analysis, the adjusted odds ratio along with a 95% confidence interval were determined. Explanatory variables whose *p* value less than 0.05 were selected as significant factors.

**Results:**

The overall uptake of routine medical checkups for common noncommunicable disease was 35.3% (95% CI: 32.34–38.26). Moreover, being married [adjusted odds ratio (AOR)=2.60, 95% CI=1.42–4.76], income level less than 7071 (AOR=3.05, 95% CI=1.23–10.05), absence of chronic medical disease (AOR=0.40, 95% CI=0.18–0.88), good provider commitment (AOR=4.80, 95% CI=1.63–14.05), drinking alcohol (AOR=0.35, 95% CI=0.19–0.65), and poor perception of health status (AOR=2.1, 95% CI=1.01–4.44) were the significant factors.

**Conclusion:**

The uptake of routine medical checkups was found to be low, owing to marital status, level of income, perception of health status, drinking alcohol, the absence of chronic medical conditions, and the availability of committed providers, which needs intervention. We recommend using committed providers for noncommunicable diseases and considering fee waivers for healthcare professionals to increase uptake of routine medical checkups.

## Introduction

Highlights

- Regular medical checkups are one of the tried-and-true methods for detecting and treating noncommunicable diseases.
- Despite a high prevalence of noncommunicable diseases, there is insufficient evidence of routine medical checkups being used by the general population or healthcare professionals in particular in low-and middle-income countries.
- The overall uptake of routine medical checkups for common noncommunicable disease was 35.3% (95% CI: 32.34–38.26).

Noncommunicable diseases (NCDs), which include cardiovascular diseases, cancer, diabetes, and chronic respiratory illnesses, are the top four global causes of mortality and disability, accounting for more than 70% of all deaths worldwide^1^,^2^. In 2019, 7.9 million deaths and 187.7 million disability-adjusted life years were attributable to preventable dietary risk factors^3^,^4^. The problem of death due to noncommunicable diseases is worse in low-income and middle-income countries, where 77% of deaths are due to NCDs^5^.

According to the WHO country profile of Ethiopia in 2016, around 39% of deaths are attributed to NCDs that can be prevented by early screening and medical checkups^6^. Among the reported deaths, cardiovascular diseases account for 16% of all deaths, which are highly preventable by providing counselling services and early screening for modifiable behavioural risk factors^6^. A subnational country analysis for the Global Burden of Disease study conducted in 2019 in Ethiopia showed NCDs are among the leading causes of death for all regions and cities^7^.

Routine medical checkups (RMC) are one or more visits with a healthcare provider for the primary purpose of assessing individuals’ overall health status and risk factors for a disease that may be prevented by early intervention^8^. Common noncommunicable diseases share common preventable behavioural risk factors, mainly tobacco use, which accounts for over 7.2 million deaths every year; an unhealthy diet, whereby around 4.1 million annual deaths have been attributed to excess salt intake; a lack of physical activity, which accounts for about 1.6 million deaths annually; and the harmful use of alcohol, which causes an estimated 3.3 million deaths globally^8^.

RMC utilization was associated with a variety of factors, including sociodemographic or predisposing factors such as age, sex, level of education, marital status, profession, etc.^9^,^10^, enabling factors like level of income, availability of health insurance, knowledge and attitude towards RMC^11^, health services related factors such as availability of regular medical checkup service, availability of committed service provider, and influence of healthcare professionals^10^,^12^, and individual need-related characteristics such as health concerns, presence of comorbidities, trust in the service, and healthy behaviours (smoking, drinking, chat, and physical exercise)^13^–^15^, were all identified as determinant factors among the general population.

NCD detection and prevention requires opportunistic case finding to identify potential risk factors^16^. RMC allows clients and clinicians discussion about the modifiable behavioural, lifestyle, and environmental related risk factors^14^,^17^. Despite an evidence that the national NCDs prevention and control strategy has been implemented in Ethiopia in recent years, the magnitude of NCDs is increasing^7^. According to the Ethiopian health sector transformative plan yearly performance report, only a small number of people were checked for common NCDs in the country in 2020, indicating the existence of a hidden barrier to using the service^18^.

There is a paucity of evidence regarding the uptake of RMC at the population level in general and among healthcare providers in particular in the NCD-pervasive city of Addis Ababa. Factors that hinder the uptake of RMCs among healthcare providers are not identified. Therefore, the aim of this study was to determine the uptake of routine medical checkups for common NCDs and their associated factors among healthcare workers at selected public hospitals in Addis Ababa, Ethiopia, in 2022.

## Methods

### Study area and population

The study was conducted in Addis Ababa at randomly selected public hospitals. Addis Ababa is the capital city of Ethiopia, which is located in the central part of the country. According to the 2019/2020 health sector transformation plan annual performance report, Addis Ababa has a total of 13 functional government-owned hospitals, 98 functional government health centres, 978 private clinics, and 30 private hospitals. The health facility coverage in the city was almost 100%^19^. The study was conducted from 1 April to 15 June 2022. The work has been reported in line with the STROCSS criteria^20^. A facility-based cross-sectional study design was employed among healthcare workers who were currently working at randomly selected public hospitals as the sample population.

### Eligibility criteria

Healthcare workers from all departments in the government-owned public hospitals who have been working at the selected facilities for more than 6 months were included in the study. However, healthcare providers who were on annual leave, educational leave, or maternity leave were excluded from the study. In addition, supportive and administrative staff were excluded from the study.

### Sample size determination and sampling procedure

The sample size was calculated using a single population proportion formula with the assumptions of a 95% confidence level, a 5% margin of error, and a 50% population proportion, implying that no previous research was available based on our literature review to estimate the level of RMC uptake in Ethiopia and to have the maximum sample size.


$$n=\frac{{\left(Z\frac{\alpha }{2}\right)}^{2}P\left(1-P\right)}{d^{2}}$$

Finally, adding a 10% non-response rate, the final sample size became 422.

A simple random sampling technique was used to select three hospitals from the 13 public hospitals in Addis Ababa. The sample size was proportionally assigned to each institution based on the total number of healthcare professionals (police hospital 949, armed force hospital 2200 and Saint Paul’s Hospital Millennium Medical College 3889). Finally, after obtaining a list of healthcare professionals from each hospital’s human resources department, healthcare providers who met the inclusion criteria were included in the study by a simple random selection procedure.

### Study variables and definitions

Uptake of routine medical checkup was regarded as the outcome variable, and sociodemographic factors (age, sex, marital status, level of education, profession, etc.), health service-related factors (availability of regular medical checkup service, availability of a committed service provider, influence of a health service provider or counsellor, etc.), and individual-related factors (healthcare providers’ knowledge of the service, healthcare providers’ attitudes towards the service, and trust in the service) were all taken as predictor variables.

#### Routine medical checkup

Is defined as one or more visits with a healthcare provider for the primary purpose of assessing individuals’ overall health status and risk factors for a disease, but it does not include preventive services that patients would receive for treatment of illness for a specific injury or condition^14^.

#### Common noncommunicable diseases

In this study, common noncommunicable diseases included cardiovascular disease, cancer, chronic respiratory tract disease, and diabetes.

#### RMC for common NCDs

An individual is considered to have received RMC if he or she receives a medical checkup at least once a year for the four common NCDs.

#### Knowledge of RMC

This is the level of understanding of RMC. It was measured by asking 13 questions. The basis for the classification of good and poor knowledge in RMC was based on the mean of each score. Individuals who scored at or above the mean were considered to have good knowledge of RMC.

#### Attitude toward RMC

This is how you perceive, feel, or think about RMC. It was measured by asking 12 questions. The basis for the classification of good and poor attitudes towards RMC was based on the mean of each score. Individuals who scored at or above the mean were considered to have a good attitude towards RMC.

### Data collection and analysis

The data were collected using self-administered, structured, and pretested questionnaires. The questionnaire has five parts which was adapted and modified from different literature. The first part assesses the general sociodemographic characteristics^10^,^21^; the second part assesses the respondents’ knowledge and attitude toward routine medical checkups^9^,^11^; and the third and fourth parts assess health service-related factors and the respondent’s experience with routine medical checkups, respectively^10^,^21^. The experience of respondents towards RMC includes whether they have received RMC or not, the frequency, the reason for a checkup, and the reason for not receiving RMC if they do not receive the service. The questionnaire was prepared in English.

The validity and reliability of the instrument’s initial content were evaluated by subject-matter experts, including researchers and medical educators. Cronbach’s alpha was 0.74 value, so reliability was not violated^22^. We used different scoring system for each factor for instance predisposing factors were identified through questions about age (in years), sex (female=1 or male=0), marital status (married=2, single=1, divorced=0), education (college diploma=2, degree=1, masters and above=0), trust in the service (yes=1 and no=0), and belief in the importance of routine checkups (yes=1 and no=0). Enabling resources questions included monthly income (<7071=2, 7072=1, 11306+=0), and availability of health insurance (had health insurance=1 and did not have health insurance=0). The perception and attitude questions were prepared using a five-point Likert scale (strongly disagree, disagree, neutral, agree, and strongly agree).

To ensure the completeness and consistency of the data, it was collected by three trained data collectors who have bachelor’s degrees in nursing and public health. One day of training was provided for data collectors regarding the content of questionnaires, how to select the sample and how to fill out the structured questionnaires, the way of communicating with research participants, and ethical issues to be considered throughout the whole study period.

Data entry was made using Epi-data and exported to STATA version 15 for cleaning and analysis. Descriptive statistical summary measures such as frequencies, cross-tabs, and the χ^2^ test were used for describing the study variables. The bivariate logistic regression analysis was performed to identify possible candidate variables for the multivariable logistic regression analysis. During the bivariate analysis, factors with *p* values less than 0.25 were included in the multivariable logistic regression analysis, as suggested by Hosmer and Lemeshow^23^. The final multivariable logistic regression model result was expressed in terms of adjusted odds ratios (AOR) and 95% CI. Eventually, explanatory variables with *p* values less than 0.05 were selected as significant factors.

### Patient and public involvement

Throughout the data collection period, respondents, including doctors, nurses, midwives, etc., provided free support and advice for the researchers related to ethical issues and advice on how to share our findings with a wide audience in a way the public can understand. However, no patient was involved in this study.

## Results

### Sociodemographic characteristics

A total of 397 respondents participated in the study, giving it a response rate of 94.1%. The mean age of respondents was 32.7 years (range=20–54, SD=6.16). Most of the respondents were female 225 (56.7%). About 190 (47.8%) respondents were married; about 184 (46.4%) respondents were nurses; and 62 (15.6%) were medical doctors. In terms of their educational status, 294 (74.1%) had bachelor’s degrees, and 28 (4.5%) had a specialty level of education. About 174 (48.8%) participants were juniors in their work experience. Almost all respondents were full-time employees of government-owned hospitals. Only 71 (17.9%) individuals were beneficiaries of the health insurance programme (Table 1).

**Table 1:** The socioeconomic characteristics of respondents in Addis Ababa, 2022 (*n*=397)

Variable	Frequency	Percent (%)
Sex of respondent		
Male	172	43.3
Female	225	56.7
Age		
20–24	30	7.6
25–29	96	24.2
30–34	112	28.2
35–39	102	25.7
40+	57	14.4
Marital status		
Single	165	41.5
Married	190	47.9
Divorced/widowed	42	10.6
Level of education		
Diploma	24	6.0
Degree	294	74.1
Masters and above	79	19.9
Profession		
GP/specialist	62	15.6
Health officer	24	6.1
Nurse and midwifery	225	56.4
Pharmacist	31	7.8
Laboratory	19	4.8
Anaesthetist	23	5.8
Radiographer	13	3.3
Work experience		
0–5 years	213	53.7
6–10 years	124	31.2
>10 years	60	15.1

### Knowledge of healthcare professionals regarding routine medical examinations

**Figure 1:**
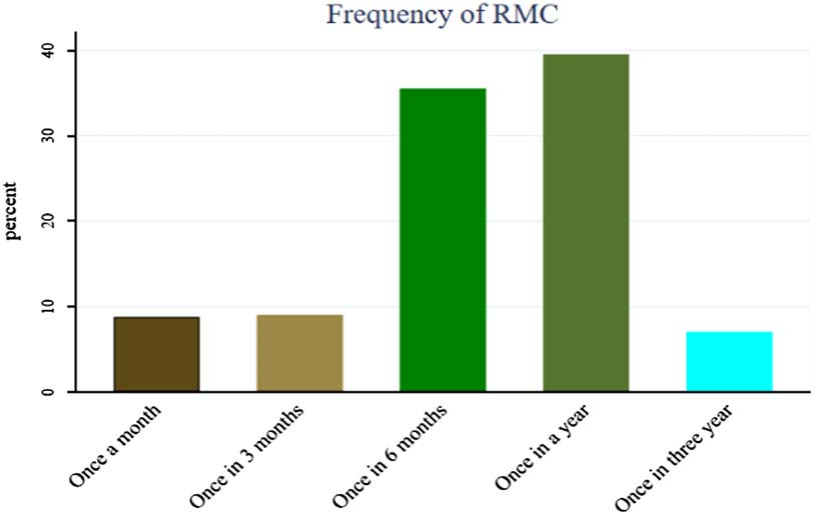
Healthcare providers awareness on the frequency of RMC in Addis Ababa, 2022. RMC, routine medical checkup.

Around 345 (86.90%) of the respondents had information about RMCs. A majority 302 (76.1%) of respondents mentioned all populations are eligible for routine medical checkups, nine (2.3%) mentioned only sick individuals should go for RMCs, 22 (5.5%) and 55 (13.9%) mentioned individuals aged 18–65 years, and those individuals who have NCDS are eligible for RMCs for common NCDs. Respondents were also asked about the main components of RMC; of the total, 355 (89.4%) and 329 (82.9%) mentioned blood pressure measurement and blood sugar checks as the main components of routine medical checkups. Regarding the frequency of RMC, most of the study respondents 157 (39.6%) mentioned that RMC should be sought once a year, and about 28 (7.1%) participants mentioned that RMC should be sought once every 3 years. Overall, about 165 (41.56%) of the study respondents scored at or above the mean and were categorized as having good knowledge of RMC (Fig. 1).

### Perception and attitude of healthcare providers towards routine medical checkups

Most of the study participants 288 (72.5%) agree with the statement that “RMCs can detect NCDs early,” and a significant number of participants 230 (57.9%) and 113 (28.4%) perceive that RMCs are expensive and time-consuming, respectively. About 317 (79.8%) of the participants mentioned that history-taking and physical examination are important parts of RMC. Overall, about 190 respondents (47.86%) scored at or above the mean and were categorized as having a favourable attitude towards RMCs (Table 2).

**Table 2:** Attitude of healthcare professionals towards RMC in Addis Ababa, 2022

Variables	Strongly disagree, *N* (%)	Disagree, *N* (%)	Neutral, *N* (%)	Agree, *N* (%)	Strongly agree, *N* (%)
RMCs can detect NCDs early	42 (10.6)	24 (6.1)	43 (10.8)	99 (24.9)	189 (47.6)
RMCs are not beneficial	156 (39.3)	143 (36.0)	20 (5.0)	55 (13.9)	23 (5.8)
RMCs are expensive	54 (13.6)	78 (19.7)	35 (8.8)	129 (32.5)	101 (25.4)
RMCs are time-consuming	54 (13.6)	132 (33.3)	98 (24.7)	75 (18.9)	38 (9.6)
It is embarrassing to go for RMC while you are healthy	60 (15.1)	137 (34.5)	84 (21.2)	75 (18.8)	41 (10.3)
RMC is good for prevention than cure	5 (1.3)	19 (4.8)	40 (10.1)	183 (46.1)	150 (3.8)
RMC should be part of one’s priority activity	22 (5.5)	11 (2.8)	32 (8.1)	214 (53.9)	118 (29.7)
RMC is a necessity to check early onset of disease	65 (16.4)	28 (7.1)	24 (6.1)	175 (44.1)	105 (26.5)
As a health professional I think I should not go for RMC	228 (57.4)	85 (21.4)	23 (5.8)	36 (9.1)	25 (6.3)
Abnormal findings in an RMC mean the person is suffering from disease	193 (48.6)	84 (21.2)	57 (14.4)	44 (11.1)	19 (4.9)
Normal findings in RMC mean that the is free from disease	201 (50.6)	67 (16.9)	64 (16.1)	42 (10.6)	23 (5.8)
History and physical examination are important part of RMC	39 (9.8)	22 (5.5)	19 (4.8)	66 (16.6)	251 (63.2)

NCD, noncommunicable diseases; RMC, routine medical checkup.

### The needs of individuals and modifiable NCD risk factors among study respondents

**Figure 2:**
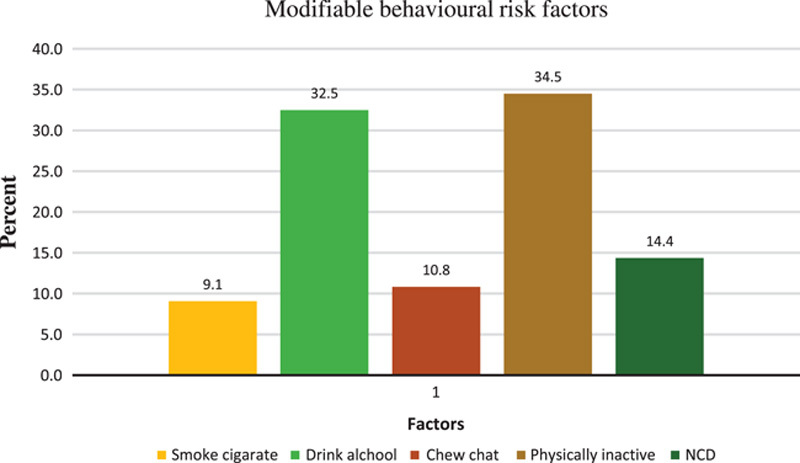
Modifiable behavioural risk factors among healthcare providers in Addis Ababa, 2022.

Among the most common modifiable NCD risk factors, about 36 (9.1%) of respondents smoked cigarettes, and 64 (16.1%) mentioned they drank three or more bottles of alcohol per day. Chat chewers comprised ~43 (10.8%) of all respondents. A significant number of individuals, 137 (34.5%), reported that they are physically inactive, with only 246 (61.9%) of respondents engaging in moderate physical activity outside of work for at least 20–30 min on 4 out of the 5 days of the week, and 274 (69.2%) of respondents using alternative means of transportation, such as walking instead of a taxi and using stairs instead of elevators. Diabetes was mentioned by 23 (40.35%) and hypertension was mentioned by 17 (29.8%) of the total 57 (13.4%) participants. Overall, only 79 (19.9%) of the participants rated their health status as “very good” (Fig. 2).

### Health services and related factors for uptake of RMC

Of the total, 324 (81.6%) explained that RMC is available in the nearby health facilities, among which 296 (74.6%) mentioned that the health facilities provide counselling services on modifiable behavioural risk factors during RMC. According to the study’s findings, only 317 (79.9%) of health facilities had a PAP smear, and 333 (83.9%) had a weight scale. Of the total, 362 (91.2%) participants had trust in the provided health service, and 79 (19.9%) respondents rated the provided service as poor. About 51 (87.2%) mentioned the performance of healthcare professionals as not committed. The most common reasons for low service rates and low commitment of healthcare professionals were a lack of services (29.3%) and a lack of service providers (33.5%), respectively (Table 3).

**Table 3:** Available services during RMC in nearby health facilities, Addis Ababa, August 2022

Variables	Frequency	Percent (%)
RMC available in nearby facilities		
Yes	324	81.6
No	73	18.4
Counselling service during RMC		
Yes	296	74.6
No	101	25.4
Availability of chemistry machine		
Yes	343	86.4
No	54	13.6
Availability of CBC machine		
Yes	344	86.6
No	53	13.4
Availability of BP measurement		
Yes	337	84.9
No	60	15.1
Availability of blood sugar measurement		
Yes	331	83.4
No	66	16.6
Weight Scale		
Yes	333	83.9
No	64	16.1
Echocardiography		
Yes	318	80.1
No	79	19.9
Availability of colonoscopy		
Yes	318	80.1
No	79	19.9
Availability of PAP smear exam		
Yes	317	79.9
No	80	20.1

BP, blood pressure; CBC, complete blood count; RMC, routine medical checkup.

### Uptake of routine medical checkups by healthcare professionals

**Figure 3:**
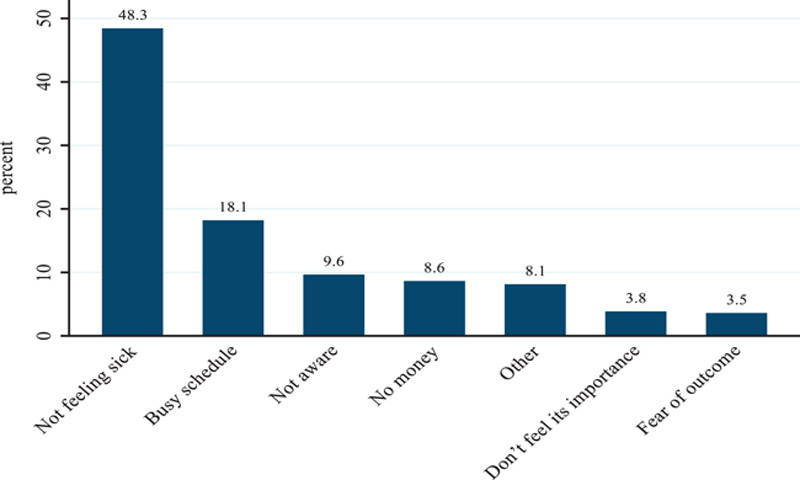
Reason for not going for RMC among healthcare providers in Addis Ababa, 2022. RMC, routine medical checkup.

Only 269 (67.8%) of respondents have ever undergone routine medical checkups, of which only 155 (39.4%) have received RMC in the past 12 months prior to the study. The majority of respondents 101 (65.2%) went for RMC, and about 23 (14.8%) went for blood pressure measurement. The main reason for not going to RMC was “I was not feeling well,” mentioned by 121 (50%) of the respondents. About 269 (67.8%) have ever participated in routine medical checkups. Overall, 140 (35.3%) (95% CI: 32.34–38.26). Health professionals were categorized as taking RMC for common NCDs once every 12 months (Fig. 3).

### Multivariable analysis of factors associated with RMC

According to a multivariable logistic regression model, married health professionals were 2.6 (AOR=2.6, 95% CI=(1.42–4.76) times more likely to use RMC than single health professionals. When compared with healthcare workers earning less than 7071 Ethiopian Birr, those making more than 11305 Ethiopian Birr per month had more than three times better odds of using RMC (AOR=3.05, 95% CI=1.23–10.05). However, healthcare professionals without a chronic noncommunicable disease were 60% (AOR=0.40, 95% CI=0.18–0.88) less likely to uptake RMC than those with a chronic noncommunicable disease. Individuals who do not drink alcohol were 65% (AOR=0.35, 95% CI=0.19–0.65) less likely to uptake RMC. Healthcare providers who had a negative self-perception of their health status were 2.1 times more likely to use RMC (AOR=2.1, 95% CI=1.01–4.44). Furthermore, respondents who mentioned the availability of committed care providers in nearby facilities were 4.80 (AOR=1.63–14.05) times more likely to uptake RMC. The result was statistically significant at a *P* value less than 0.05 (Table 4).

**Table 4:** Multivariable analysis of factors associated with uptake of RMC among healthcare professionals in Addis Ababa, 2022

	Uptake of RMC			
Variables	Yes (%)	*N* (%)	COR (95% CI)	AOR (95% CI)
Sex				
Male	68 (39.5)	104 (60.5)	1	1
Female	72 (32.0)	153 (68.0)	0.72 (0.48–1.09)*	1.01 (0.61–1.69)
Marital status				
Single	47 (28.5)	118 (71.5)	1.00	
Married	83 (43.7)	107 (56.3)	1.94 (1.25–3.03)**	2.60 (1.42–4.76)**
Divorced/separated	10 (23.8)	32 (76.2)	0.78 (0.36–1.72)	0.49 (0.16–1.45)
Profession				
Nurse/midwifery	81 (36.0)	144 (64.0)	1	1
Health officer	11 (45.8)	13 (54.2)	1.50 (0.64–3.51)	0.52 (0.17–1.55)
General practitioner	28 (51.3)	19 (48.7)	1.87 (0.94–3.70)* 0.07	1.00 (0.39–2.58)
Specialist/subspecialist	8 (34.8)	15 (65.2)	0.95 (0.39–2.33)	0.08 (0.02–0.33)
Pharmacy	4 (12.9)	27 (87.1)	0.26 (0.09–0.78)**	0.08 (0.02–0.30)
Laboratory	3 (15.8)	16 (84.2)	0.33 (0.09–1.18)*	0.07 (0.01–0.38)
Anaesthetist	6 (26.1)	17 (3.9)	0.63 (0.24–1.65)	0.49 (0.15–1.53)
Radiologist	7 (53.9)	6 (46.1)	2.0 (0.67–6.38)*	0.38 (0.076–0.88)
Level of income				
Less than 7071	42 (30.9)	94 (69.1)	1	1
7072–11, 305	63 (33.3)	126 (66.7)	1.12 (0.69–1.79)	0.94 (0.47–1.89)
11, 306+	35 (48.6)	37 (51.4)	2.11 (1.17–3.81)*	3.52 (1.23–10.05)**
Knowledge towards RMC				
Good	92 (39.7)	140 (60.3)	1.60 (1.0–2.45)**	1.07 (0.61–1.88)
Poor	48 (29.1)	117 (70.9)	1	1
Attitude towards RMC				
Favourable	84 (44.2)	106 (55.8)	2.14 (1.40–3.25)***	1.45 (0.80–2.62)
Unfavourable	56 (27.1)	151 (72.9)	1	1
Availability of NCDs				
Yes	28 (49.1)	29(50.9)	1	1
No	112 (32.9)	228 (67.1)	0.51 (0.29–0.89)**	0.40 (0.18–0.88)**
Drink alcohol				
Yes	57 (44.2)	28 (55.8)	1	1
No	83 (31)	229 (69.0)	0.56 (0.3–0.87)***	0.35 (0.19–0.65)***
Availability of RMC in nearby facilities				
Available	126 (38.9)	198 (61.1)	1	1
Not available	14 (19.2)	59 (80.8)	0.37 (0.20–0.69)**	0.56 (0.23–1.33)
Availability of counselling service				
Yes	120 (40.5)	176 (59.5)	1	1
No	20 (19.8)	81 (80.2)	0.36 (0.21–0.62)***	0.47 (0.21–1.05)
Rate on quality of service				
Good	122 (38.4)	196 (61.6)	1	1
Poor	18 (22.8)	61 (77.2)	0.47 (0.27–0.84)**	0.70 (0.34–1.46)
Staff commitment to RMC				
Committed	134 (38.7)	212 (61.3)	4.74 (1.97–11.4)***	4.80 (1.63–14.05)**
Not committed	6 (11.8)	45 (88.2)	1	1
Perception on health status				
Good	105 (31.8)	225 (68.2)	1	1
Poor	35 (52.2)	32 (47.8)	2.34 (1.38–3.99)**	2.1 (1.01–4.44)**

AOR, adjusted odds ratio; COR, crud odds ratio; NCD, noncommunicable diseases; RMC, routine medical checkup.

* Significant at *P* < 0.25.

** Significant at *P* < 0.05.

*** Significant at *P* < 0.001.

## Discussion

RMC are intended to identify risk factors and early signs of disease, as well as prevent future illness through early intervention. Depending on the patient’s age and sex, RMCs are recommended to reduce premature mortality, including counselling, physical examination, and related services. Therefore, this study was intended to assess the uptake of RMC services by healthcare professionals working at selected governmental health facilities in Addis Ababa, Ethiopia.

In this study, the majority of the study participants had information about RMC, which was in line with a study conducted in south-west Nigeria^24^ in which 100% of health professionals had information about RMC, which may be related to the fact that health professionals have various sources of information, including medical books, medical schools, and high-impact medical journals. However, the overall level of knowledge was lower than a study conducted in Benin^21^ and Nigeria^24^, which may be due to the unavailability of standardized national RMC guidelines, specifically, the frequency, recommended age groups, and recommended services for RMC. About half of the study participants had a favourable attitude towards RMC, which was consistent with a study conducted in Cross River State, Nigeria^25^. The majority of study participants thought RMC should be done once a year, then every 6 months. However, a similar study conducted in Nigeria among healthcare provider respondents mentioned that RMC should be conducted once every 6 months, followed by one every year^26^. The possible explanation for the discrepancy may be related to the national recommendation for conducting RMCs.

In this study, about 67.8% of healthcare professionals have ever participated in the RMC, which is higher than a study conducted in Uganda’s general population, which was 43.4%^27^. The difference may be due to healthcare professionals in our study being more aware of the importance of RMC and the high accessibility of health facilities for healthcare professionals. However, the overall uptake of RMC once every twelve months was only 35.3%, which was found to be low.

Studies were limited to determine the uptake of RMC once a year among healthcare providers and the general population. But the level of uptake was higher than in a study conducted on health science students in Saudi Arabia (25.5%), Nigeria (28.4%), and Vietnam (24.5%)^10^,^25^,^28^, which may be due to knowledge differences at RMC. The lower level of RMC uptake among healthcare providers might be attributed to the negligence of healthcare professionals to practice RMC despite the availability of a high level of information on the services. The study’s finding indicated that, while the majority of healthcare professionals are aware of RMC, they do not put it into practice. This means that the government must implement sensitization and guidelines for conducting RMC.

In our study, the most common medical checkups were general physical examination (72.1%) and blood pressure checkup (16.4%). Of the total female respondents, only seven (9.7%) have participated in a cervical cancer pap smear examination, and no one has participated in a breast cancer examination. The finding of the study was lower than a study conducted among Benin healthcare providers, where (28.8%)^23^ have participated in the PAP smear examination, it may be linked to the lack of required engagement of health professionals in RMC by health facilities in our context. This entails creating a formal recommendation for health workers operating in facilities to undergo RMC for common NCDs.

The marital status of healthcare professionals was significantly associated with the uptake of RMC among healthcare professionals. A similar finding was reported in a study of healthcare professionals in Edo State^23^ and a study based on secondary data in Iowa, western US, where married people were more likely to undergo RMC^29^. The possible explanation for the high uptake of RMC by married individuals may be attributed to the increase in age and availability of family persuasion to go for RMCs among married health professionals.

Individuals with higher income levels were found to be more likely to uptake RMC than those with a lower level of income. Previous research has found that the cost of service has a significant impact on the uptake of RMC among healthcare professionals^29^,^30^. This could be explained by the fact that individuals with lower incomes may not seek RMC because of the expense, underscoring the importance of contemplating the provision of exempted RMC services for healthcare professionals working in various health institutions.

Furthermore, individuals who do not drink alcohol were 65% less likely to uptake RMC, indicating that those who drink alcohol were more likely to uptake RMC compared with their counterparts. It was consistent with research findings from Rhode Island and the United States^31^,^32^. This finding can be explained by the fact that alcoholic drinkers may think professionally that they are at high risk of acquiring common NCDs attributed to alcohol drinking.

The uptake of RMC was further influenced by the availability of chronic NCDs in the healthcare system. A cross-sectional study conducted in Nigeria^23^ and Taiwan^33^ indicated that being managed for medical conditions was significantly associated with the uptake of routine medical checkups. The consistency of findings could be attributable to the fact that individuals with known medical conditions periodically go for medical checkups to determine the progress, improvement, and effect of medications.

Another factor positively associated with the uptake of RMC was the availability of committed healthcare providers in the nearby health facilities, which is consistent with a study finding from different literature^34^,^35^. This might be due to the fact that the availability of highly competent and compassionate healthcare providers in the health facilities can motivate the receiver to engage in periodic medical examinations. The discovery could be viewed as a warning sign that building good hospitality, training, and deploying highly respectful and compassionate healthcare staff are the foundation for inspiring care receivers to participate in RMC for common NCDs. The discovery can be applied to the broader population.

The uptake of RMC further influenced the healthcare providers’ perceptions of their health status. Healthcare professionals who perceived that their health status was poor were more likely to go for RMC than their counterparts. The outcome was consistent with a study finding from other literature^36^,^37^. This study’s findings indicated that individuals’ needs to determine whether they seek RMCs for common NCDs.

### Strengths and limitations of the study

The strength of this study relies on the inclusion of different healthcare professionals from a representative sample of government-owned hospitals. However, the study is not without limitations. First, the study design was insufficiently robust and may have failed to distinguish between cause and impact relationships. Besides, the study does not use validated tools and does not include healthcare professionals from the private sector due to time and budget constraints, which will be a feature of future research directions to include the private sector and rural healthcare professionals and explore the major barriers and facilitators in detail. The findings of the study are not generalizable to the general population since significant knowledge and accessibility differences may affect the uptake in the general population. The final but not least weakness of this study is that the findings may be influenced by social desirability bias.

## Conclusion

In conclusion, the uptake of RMC for common NCDs among healthcare providers was found to be low. The majority of the study participants were found to have a favourable attitude and information towards RMC. However, a significant number of healthcare professionals do not have the required knowledge about the expected frequency of RMC, eligible individuals for RMC, or the main reason for uptake of RMC. Even if the majority of the study participants had information about RMC, it was not practiced by a significant number of professionals.

The finding of the study implies that RMC and counselling services were mentioned as available in nearby facilities, but an undeniable number of professionals questioned the quality of service and staff commitment to providing the service. A significant number of healthcare professionals were found to have modifiable behavioural risk factors, including cigarette smoking and sedentary lifestyles. Furthermore, marital status, monthly income, the presence of NCDs, the commitment of healthcare providers in nearby facilities, and poor perceived health status all had a significant impact on RMC uptake among healthcare professionals.

Therefore, the Ethiopian Ministry of Health should conduct annual sensitization campaigns and medical examinations. We also recommend using committed providers and considering fee waivers for healthcare professionals to promote acceptance of routine medical checkups.

## Ethical consideration

Ethical approval was obtained from the Yanet College research and ethical review board with approval number YEC/032/22. Then a permission letter was secured from each selected health facility’s higher officials. Written informed consent was also obtained from each study participant after detailed information was provided to each individual. The ethical principles outlined in the Declaration of Helsinki guided the entire research process, which states that “it is the physician’s or researcher’s responsibility to promote and protect the health, well-being, and rights of study participants, including those who participate in medical research”^38^. Moreover, all the study participants were informed that the participation was voluntary and of the potential benefits, confidentiality, and possibility of withdrawing from the interview at any time.

## Consent

Written informed consent was obtained from each study participant before an interview.

## Source of funding

The study has no funding source.

## Author contribution

G.K.G.: conceptualization, data curation, methodology, supervision, and writing, review, and editing. M.A.: conceptualization, formal analysis, and writing original draft. G.K.: Data curation, formal analysis and visualization. A.S.: Data curation and writing original draft. D.B.: Data curation, supervision, and visualization.

## Conflicts of interest disclosure

The authors declare that they have no competing interests.

## Research registration unique identifying number (UIN)

Name of the registry:

http://www.researchregistry.com

Unique identifying number or registration ID:

researchregistry8295

## Guarantor

All authors will take responsibility for the work, access to data, and decision to publish.

## Data availability

The datasets used and/or analyzed during the current study are available from the corresponding author.

## Provenance and peer review

Not commissioned, externally peer-reviewed.
